# Adaptive Software Defined Equalization Techniques for Indoor Visible Light Communication

**DOI:** 10.3390/s20061618

**Published:** 2020-03-14

**Authors:** Radek Martinek, Lukas Danys, Rene Jaros

**Affiliations:** Department of Cybernetics and Biomedical Engineering, Faculty of Electrical Engineering and Computer Science, VSB–Technical University of Ostrava, 17. listopadu 15, 708 33 Ostrava, Czech Republic; radek.martinek@vsb.cz (R.M.); lukas.danys@vsb.cz (L.D.)

**Keywords:** feed-forward software defined equalization (FSDE), visible light communication (VLC), software-defined radio (SDR), multistate quadrature amplitude modulation (M-QAM), adaptive equalizers, QR decomposition based recursive least squares (RLS)

## Abstract

This paper focuses on a channel feed-forward software defined equalization (FSDE) of visible light communication (VLC) multistate quadrature amplitude modulation (M-QAM) based system, implemented in the LabVIEW programming environment. A highly modular platform is introduced; the whole experiment is simulated in software and then thoroughly explored and analyzed during practical measurements in the laboratory, simulating real-world situations. The whole platform is based on modified National Instruments software defined radios (NI SDR) and a commercially available Philips light source, often used in Czech government institutions. Three FSDE algorithms were tested: least mean squares (LMS), normalized least mean squares (NLMS), and QR decomposition based RLS (QR-RLS). Based on measurements, QR-RLS provides the best results, improving measured values by up to 10%. The experiments also show that the simulated results are very similar to real measurements, thus proving the validity of the chosen approach. The whole platform manages to improve measured data simply by making changes to the software side of the testing prototype.

## 1. Introduction

**Figure 1:**
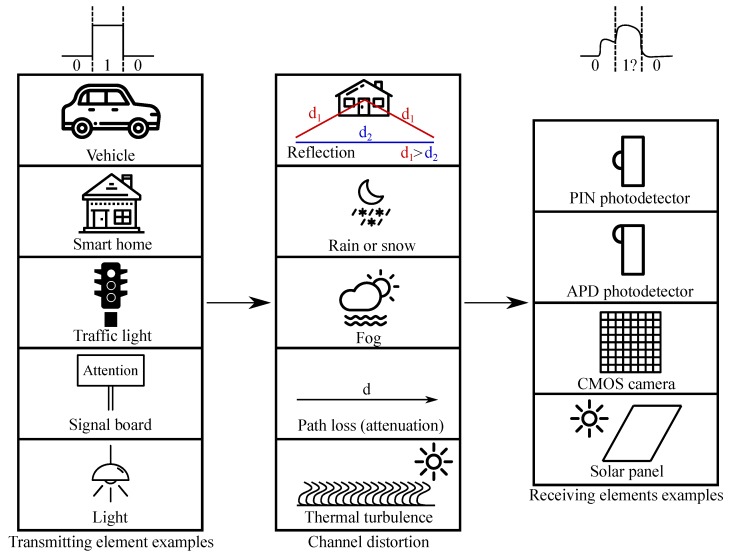
Examples of various impairments in visible light communication systems. Examples cover all scenarios from vehicle-to-everything (V2X) to machine-to-machine communication (M2M).

In wireless and visible light communications (VLC), data are susceptible to interference caused by variations in the transmission channel. In the ideal communication channel model, the signal propagates directly from the transmitter to the receiver. However, the ideal model is practically unreachable as a number of reflected signals caused by various obstacles influence the whole platform. As their time of arrival (ToA) at the receiver varies in comparison to the direct wave, they cause a phenomenon called multipath propagation. In addition, various atmospheric effects, such as rain, fog, or even temperature turbulence can cause additional noise; see [1,2,3,4] (Figure 1).

Figure 1 represents various scenarios of visible light communication systems. The left side represents various transmitting sources (represented by physical solutions), which are often explored by scientists. The center column represents examples of various channel impairments, which influence the transmitted signal. The right column presents examples of receiving solutions, spanning from conventional PIN photodetectors [5,6,7,8,9] to pricier avalanche photo diodes (APDs) [10,11,12], advanced CMOS cameras [13,14,15,16,17], and inexpensive alternatives in solar panels [18,19,20,21,22].

As a result of signals being delayed differently (due to multipath propagation), so-called intersymbol interference (ISI) occurs on the receiving side. ISI is a major concern in visible light communication, as the medium (light) can be reflected by various surfaces [23]. It can be also caused by the position of LEDs or the bit interval [24]. Intersymbol interference is a situation when one symbol (or state) interferes with other symbols, thus increasing the error rate and leading to the lower reliability of the whole platform. Adaptive equalization is one of the ways to overcome intersymbol interference; see [25,26,27,28,29]. Error correction codes can be also used to alleviate ISI [24].

Currently, the requirements for quality of RF and VLC systems are slowly increasing, and even higher data rates are desired. However, by increasing the number of symbols transmitted during a period of time, the length of said symbols is shortened; therefore, any delayed pulse that arrives outside the set interval causes significant distortion of the transmitted data. Modern microwave links can use up to 4096-QAM [30]; however, the signal quality requirements increase significantly for modulation formats with a large number of states.

Wang et al. in 2015 [31] used RLS based adaptive equalization for RGB-LED based wavelength division multiplexing (WDM) VLC system. They compared RLS algorithm with the modified cascaded multi-modulus algorithm (M-CMMA) and concluded that the RLS algorithm can outperform it by a Q factor of 1 dB. Their results showed that an adaptive equalization scheme for indoor high-speed VLC systems provides benefits and is feasible. Wang et al. in 2014 [32] proposed a hybrid time-frequency adaptive equalization algorithm, which combines frequency domain equalization (FDE) and decision-directed least mean squares (DD-LMS). By using this hybrid system, they reached improvements in performance. They concluded that their system reached the highest data rate ever (until 2014) by using a single commercially available RGB-LED in a VLC system. Sirvi and Tharani in 2016 [33] tested NLMS equalization. They concluded that the bit error ratio (BER) was vastly improved by employing NLMS based equalization. Akande et al. in 2017 [34] tested a linear adaptive least mean squares fractionally spaced equalizer to mitigate ISI and jitter. In their work, LMS managed to outperform the conventional symbol spaced equalizer (SSE) with three times better results. Moreover, LMS was insensitive to timing jitter. Zhang et al. in 2016 [35] tested an LMS algorithm against a novel scalar modified cascaded multi-modulus algorithm (S-MCMMA). Based on their work, both algorithms had similar results while deployed in a pulse-amplitude modulation (PAM)based system. Mitra and Bhatia in 2017 [36] presented their work focusing on Chebyshev-NLMS based pre-distorter LED compensation in non-orthogonal multiple access (NOMA)-VLC. Their work partially focused on deployment in IoT with different QoS levels in mind. Their claims were backed up by simulations. Wang et al. in 2016 [37] presented their work focusing on spectrally efficient fxrequency division multiplexing (FDM)with RLS time-domain channel estimation. RLS vastly improved the performance of the whole presented platform.

This work focuses on an adaptive equalization implementation in a previously designed multistate quadrature amplitude modulation (M-QAM) VLC system [38]. A number of adaptive algorithms are tested, such as least mean squares (LMS), normalized least mean squares (NLMS), or QR decomposition based RLS (QR-RLS). All these algorithms are tested in simulations and real-life measurements. This article mainly focuses on the error vector magnitude (EVM), modulation error ratio (MER), and BER parameters and their evaluation across different equalization algorithms. Measurements are carried out in laboratory at the Faculty of Electrical Engineering and Computer Science of VSB–Technical University of Ostrava.

**Figure 2:**
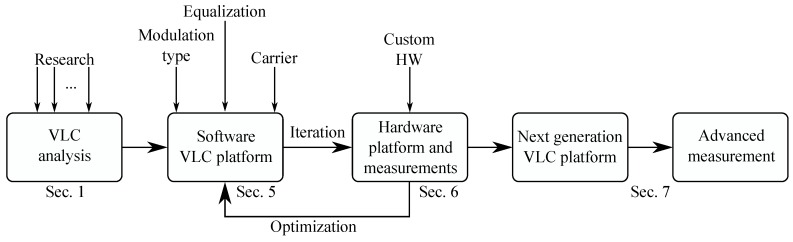
VLC prototyping research concept.

Figure 2 represents the whole prototyping process. In the beginning, research was carried out to determine the rough design of the platform. This software based simulation had to be highly modular and independent; therefore, any additional changes could be quickly tested for future deployment on hardware. Feedback from hardware could be then used for further optimization of the simulation platform, which again would influence the future form of the hardware design. Each major “release” of the hardware platform could be also further used for advanced measurements in the BROADBAND${}^{\mathrm{LIGHT}}$ testing polygon situated next to the Faculty of Electrical Engineering and Computer Science (described more in detail by Baros et al. [39]) or real-world scenarios. The whole “iteration <–> optimization” process was mainly used during the estimation of the ideal equalization parameters and the training sequence length.

## 2. Advances in Visible Light Communications

**Figure 3:**
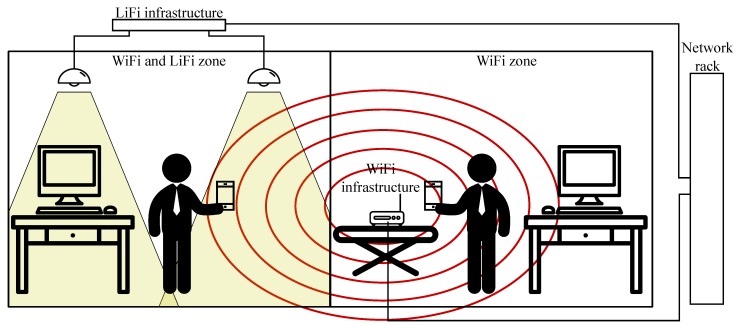
Pure WiFi environment vs. WiFi and light fidelity (LiFi) zone. WiFi signals pass through individual walls, thus limiting the reusability of the channels.

Visible light communication is slowly surfacing as a fully-fledged alternative or at least a supplementary technology to today’s widespread wireless fidelity (WiFi). WiFi tends to lack free channels in highly urbanized areas or flat complexes [40]. Visible light communications provide an alternative, which does not penetrate walls, thus ensuring high reusability of the channels (Figure 3). As the technology itself operates in the free band, which is currently not limited by strict standardization, it is possible to develop practically any custom system; see [41,42,43].

Many companies focusing on the manufacturing of LED light sources or even smart devices (such as smart phones) are already exploring the possibilities of light fidelity (LiFi) or have claimed multiple patents in this field. Both major smartphone players, Samsung and Apple, are currently developing their own versions of LiFi. Samsung holds multiple patents in this field [44]. On the other hand, Apple actively cooperates with PureLifi company, based around professor Haas, who pioneered one of the first experiments with LiFi itself [45]. PureLifi is experimenting with LiFi modules integrated into laptops as well, reaching up to one gigabit [46].

In the case of conventional WiFi, the new standard called 802.11ad was recently released, and active network elements are slowly surfacing on the market. This standard moves WiFi from 5 GHz to 60 GHz, also called the mm-wave region. However, the path loss will significantly increase since it is proportional to the square of the carrier frequency. It is estimated that either the transmit power has to be increased, the distance between individual access points has to be decreased, or the whole system has to employ advanced beamforming to focus transmissions [47]. As the current regulations for 4G/5G systems have reached their ceiling, it is not likely that the transmit power will increase in the enhanced 5G or possibly 6G network. Thus, a different approach has to be chosen such that the cell size of 5G/6G will be reduced, which will inevitably lead to the increase of manufacturing costs, since the beamforming, which requires multiple transmitting/receiving antennas, will be a necessary technology. On the other side are VLC technologies with the unlicensed spectrum of approximately 350 THz. However, the VLC systems suffer from high path loss; thus, advanced optical components are practically a must to develop a long-distance system. Currently, VLC is mainly used in free space optical (FSO) wireless links, which have advanced collimators or even automatic fine-tuning capabilities and are often used as the backbone of Internet providers. Haas et al. differentiated between VLC, optical camera communications (OCC), free-space optical communications (FSO), and light fidelity (LiFi) [48]. According to him, VLC should be used for IoT or machine-to-machine communications providing ultra-reliable low latency communication (URLLC) for 5G, B5G, and Industry 4.0. OCC is similar to VLC, but can also use a display as the transmitter and a camera image sensor as the receiver. This whole concept stands on recent advances in image sensor and camera technologies, offering higher resolutions and/or frame rates. Furthermore, all the sensors are currently much smaller and can be embedded into smartphones or laptops. Other than data transmissions, this technology can be also used for localization [49]. As mentioned before, FSO links based on laser diodes are often used for backhaul connections, where optical fibers can be reliably used (or the solution would be pricy). Finally, LiFi is a completely wireless network solution, serving as a complete alternative or supplementary technology to WiFi. This technology uses visible light for receiving data and the infrared band for transmissions. The coverage of LiFi is severely limited by the illumination area of the light source.

One of the main advantages of VLC technology is that it does not interfere with machinery or medical devices. Currently, if devices in factories or medical institutions have to be online (even on a local network), they are usually connected via Ethernet or optical cables, as these devices tend to be highly sensitive to any RF interference. However, this approach has its limits, since it is not always convenient to wire movable machines. Therefore, an alternative, which lies in VLC systems or LiFi, could significantly improve the reliability or even the portability of said devices [50].

Multiple complications were encountered during the development of the previously mentioned VLC based systems. Among them is the receiver sensitivity, as most of the photodetectors were designed for fiber optics, where the beam is precisely focused on the photodiode itself. Therefore, the sensitivity tends to be low, and multiple solutions are currently being explored. Since it is practically impossible to increase the size of the photodiode itself without influencing the resulting bandwidth, many teams tend to develop optical concentrators. Putri et al. presented their own concentrator, which managed to improve the communication coverage of an on-off keying (OOK)based system by approximately 15 to 55% [51]. A fluorescent concentrator can be even used to implement a MIMO system, as presented by Mulyawan et al. [52]. Apart from different concentrators, a fly-eye receiver can be used [53] or even an advanced form of an angle diversity receiver, which is often combined with beam steering technology [54,55]. Zhang et al. [56] compared APD and single photon avalanche diode (SPAD)based receivers, which are also often used in experiments. APD showed promising results, but it tends to be pricy for mass deployment.

Beamforming is another area that is currently explored not only in VLC, but also in 5G and the new WiFi 6 [47,57,58]. This technology can significantly improve resource allocation or the reusability of frequency bands. Cen et al. presented their own VLC beamforming solution called LiBeam, which managed to improve network spectral efficiency significantly, thus increasing the throughput of the whole platform [57]. VLC integration into 5G networks was also tested by Shi et al. [59].

VLC systems and light propagation can be somewhat reliably simulated by employing ray-tracing technologies, which are currently on the rise, as GPUs with dedicated ray-tracing hardware are available. Lichtenegger et al. introduced their own ray-tracing platform for the simulation of VLC channel modeling [60].

## 3. Methods

**Figure 4:**
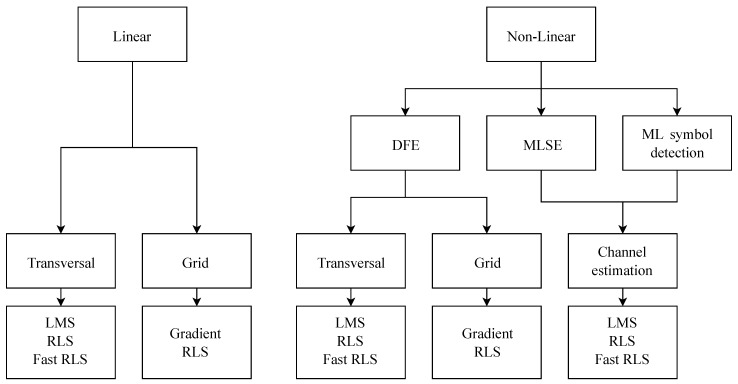
Classification of adaptive algorithms. LMS, least mean squares.

It is very important to use a suitable equalization algorithm to improve the system performance (Figure 4). Some algorithms provide very good accuracy in some areas, but cannot be used for equalization. Based on the literature, algorithms based on LMS and RLS provide very good accuracy for equalization; therefore, the LMS, NLMS, and QR-RLS algorithms were chosen for this article. The rest of this section deals with the basic mathematical description of the chosen methods. For a more detailed description, refer to the cited articles.

### 3.1. Least Mean Squares

The LMS algorithm is very often used for adaptive filtering, as it is very simple and not time demanding. This algorithm is a stochastic gradient descent adaptive method based on Wiener filtering theory, stochastic averaging, and the least squares method. Calculation of the linear adaptive algorithm coefficients is given by Equation (3), where x is the input signal, w represents the impulse characteristic of the filter, and μ is the step size. Step size μ is one of the main parameters. When the value of μ is too low, it takes a very long time to find the optimal solution of the adaptive filter. On the other hand, when the value of μ is too high, the adaptive filter becomes unstable. Therefore, it is very important to find a compromise between the speed and stability of the adaptive algorithm convergence [33,34,35].

For optimization, a random starting point is selected in the coefficient space, and then by subsequent steps, the optimum point is reached. To reach optimization, the method of steepest descent is very often used. The implementation of the LMS algorithm is basically composed of three steps. In the first step, the finite impulse response (FIR)filter output value is calculated by Equation (1). Then, the estimated error value is calculated based on Equation (2). In the last step, the values of the FIR filter weights are updated with respect to the next iteration of Equation (3) [33,34,35].

(1) $$y\left(n\right)=\sum_{i=0}^{N-1}w\left(n\right)x\left(n-i\right)=w^{T}\left(n\right)x\left(n\right).$$

(2) e(n)=d(n)−y(n).

(3) w(n+1)=w(n)+2μe(n)x(n).

### 3.2. Normalized Least Mean Squares

If the input signal is relatively high, the LMS algorithm tends to often amplify the noise instead of the effective signal. For these types of signals, the NLMS algorithm has the greatest potential. Step size μ is normalized by the energy of the input signal by Equation (4). When the input signal consists of high values, it takes more time to reach the lowest possible error value. On the other hand, when the input signal consists of lower values, it will be faster to reach the lowest possible error value [33,36].

Every iteration of the NLMS algorithm requires four different steps. In the first step, the FIR filter output value is calculated by Equation (1). Then, the estimated error value is calculated by Equation (2). In the third step, the convergence constant μ is calculated by Equation (4). In the last step, the values of the FIR filter weights are updated with respect to the next iteration by Equation (3) [33,36].

(4) $$\mu \left(n\right)=\frac{1}{x^{T}\left(n\right)x\left(n\right)}.$$

### 3.3. QR Decomposition Based Recursive Least Squares

The QR-RLS leverages the advantages of the triangulation process with good mathematical properties [37,61]. It is based on robust QR decomposition, which contains the Givens transformation. It is a numerically stable algorithm with positive definiteness [37,61].

In the first step, the filter output is calculated based on Equation (5). Estimation of QR-RLS is determined by Equation (6), where d(n) is a desired signal, $\vec{w^{T}}\left(n\right)$ is a weight vector, and x(n) stands for the reference signal. The estimated error e(t) is calculated according to Equation (6) [33,36].

(5) $$\vec{y}\left(n\right)={\vec{w}}^{T}\left(n\right)\vec{x}\left(n\right).$$

(6) $$e\left(n\right)=d\left(n\right)-{\vec{w}}^{T}\left(n\right)\vec{x}\left(n\right),$$

The filter periodically updates its weights based on Equation (7), where p(n) is the corresponding vector and R(n) represents the triangular matrix [33,36].

(7) $$\vec{w}\left(n\right)=R^{-1}\left(n\right)\vec{p}\left(n\right),$$

### 3.4. Structure of the Linear Equalizer

The coefficient vector of the equalizer $\vec{\hat{w}}$, representing the optimal values of the linear equalize, r is based on the minimum mean squared error (MMSE) estimation between the original and the distorted signals. The given coefficients are set according to the input values and calculated using various algorithms, such as LMS or RLS.

**Figure 5:**
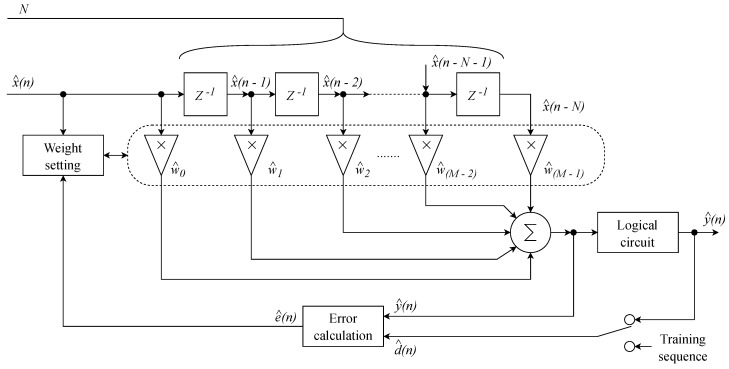
Block diagram of a linear equalizer as a linear FIRfilter with a transverse structure.

The decision circuit ensures rounding to the nearest value within the used constellation diagram. The error can be determined either based on the knowledge of the used constellation diagram or on the basis of the so-called training sequence, which is transmitted before the actual data stream (Figure 5). If a linear equalizer is used in multi-tone modulations, a separate equalizer is inserted in each output branch of the receiver.

## 4. Evaluation Parameters

Simulation, as well as measurements were carried out based on the following parameters.

BER is an important quality indicator of digital transmission systems. It is the number of bit errors per unit time; therefore, it is calculated as the number of bit errors divided by the total number of transmitted bits in one time period.

MERrepresents a relationship between the signal-to-noise ratio and error vector magnitude. It is used to evaluate the performance of the transmitter and/or receiver in systems that employ digital modulations. It is mainly influenced by channel quality and path propagation, which cause the constellation points to deviate from the ideal location.

**Figure 6:**
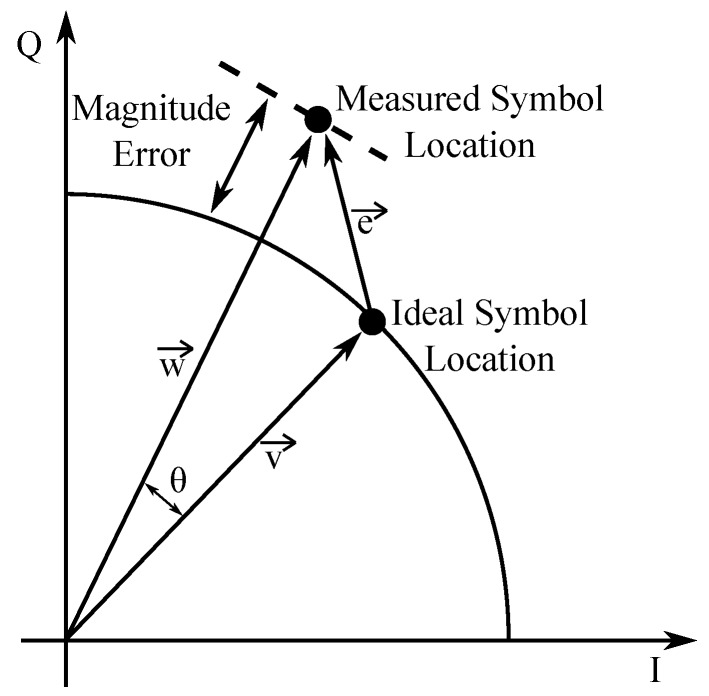
EVMin part of the IQ diagram.

EVM provides a comprehensive measure of the quality of the modulator and/or demodulator performance in the presence of various impairments (Figure 6). Various imperfections in implementation, such as carrier leakage, phase noise, shot noise, etc., cause the deviation of constellation points. EVM is used to quantify how far the points are from the ideal location. There is a one-to-one relationship between EVM and MER; however, MER is calculated from the average power of the signal.

The energy per bit-to-noise power spectral density ratio (E${}_{b}$/N${}_{0}$) is an important parameter of digital communication systems. It is basically the SNR per bit (normalized SNR measurement). It is often used to compare the BER performance of various digital modulations without the influence of the used bandwidth. SNR is periodically obtained at the receiving universal software radio peripheral (USRP) and then calculated by a computer running LabVIEW.

## 5. Simulations

The simulation platform based on virtual instrumentation was developed to estimate the results of future measurements roughly. It consisted of multiple blocks from the NI Modulation Toolkit and Adaptive Filter Toolkit. The channel itself was modeled by inserting additive white Gaussian noise block (AWGN), which simulated impairments during laboratory measurements. The results were represented not only by constellation diagrams of the transmitted/received signal, but also by multiple parameters such as EVM, BER, phase error, magnitude error, etc. Three equalization techniques were tested: QR-RLS, LMS, and NLMS.

Other than white Gaussian noise, the software was also capable of simulating the Rician fading channel or Rayleigh fading channel. These two were not tested during the first phase, but they will be useful for further measuring scenarios, such as propagation in a highly reflective environment. This software was developed as an extension of previously explored channel modeling applications [62]. Various aspects, which can be further analyzed, were thoroughly explained in the cited article.

**Figure 7:**
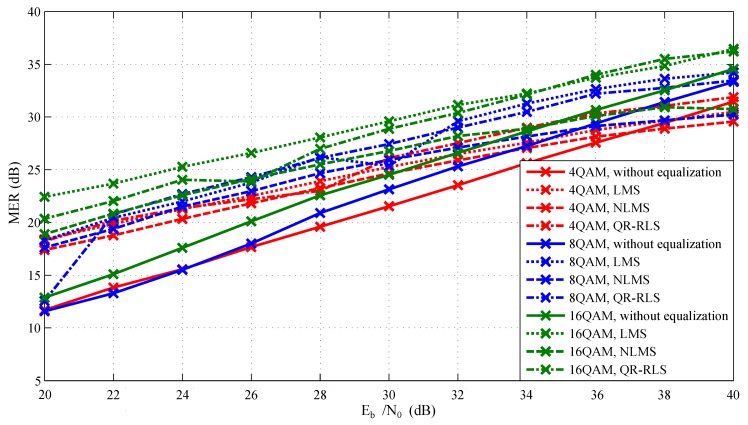
Dependence of MERon E${}_{b}$/N${}_{0}$ for lower state M-QAM modulation formats. NLMS, normalized least mean squares.

**Figure 8:**
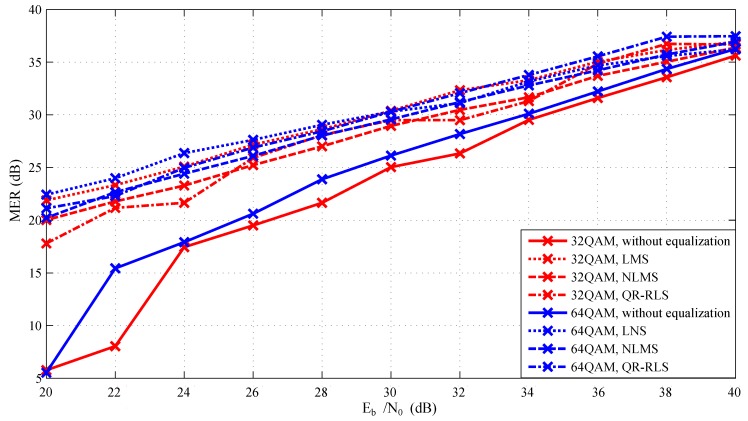
Dependence of MER on E${}_{b}$/N${}_{0}$ for higher state M-QAM modulation formats.

**Figure 9:**
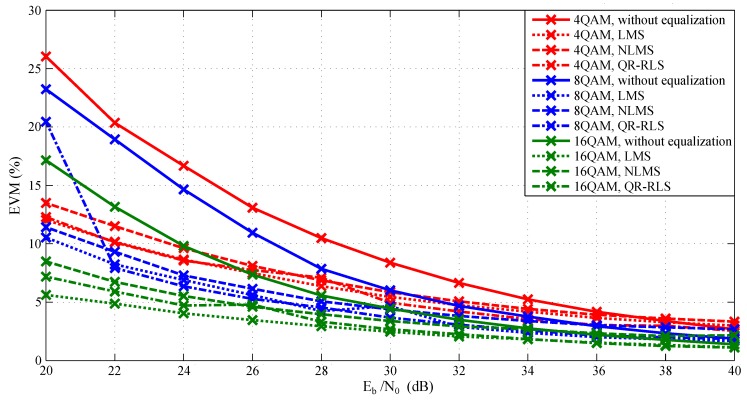
Dependence of EVM on E${}_{b}$/N${}_{0}$ during simulations.

Simulations were carried out repeatedly for each modulation format/equalization technique combination, and the E${}_{b}$/N${}_{0}$ parameter of AWGN was gradually changed from 20 to 40 dB. BER, MER, and EVM parameters were used for evaluation. Modulation order of QAM was changed from 4-QAM to 64-QAM. Figure 7 and Figure 8 represent the relationship between E${}_{b}$/N${}_{0}$ and MER. Figure 9 displays the relationship between EVM and E${}_{b}$/N${}_{0}$. Individual graphs more or less corresponded to the assumptions. Measured values corresponded to the decreasing E${}_{b}$/N${}_{0}$ since the signal itself was gradually influenced by noise. It is noticeable that equalization techniques significantly improved the MER values. They were advantageous mainly at 20 to 32 dB, where their deployment improved the reliability of the whole platform. However, their contribution was heavily influenced by the amount of noise. When the signal had higher E${}_{b}$/N${}_{0}$ values, the benefit of equalization rapidly decreased. In worst-case scenario (at 20 dB), the difference between the signal with and without the equalizer was more than 15 dB.

The whole simulation used a bandwidth of 1 MHz. The used number of transmitted symbols mainly corresponded to the performance of the measuring computer. A tradeoff between simulation performance and measurement accuracy was chosen, as the number of transmitted symbols influenced the maximal measurable order of BER. By choosing 5000 symbols, the whole simulation was able to measure at least 10${}^{-5}$ BER reliably. Anything higher than 10${}^{-5}$ was therefore rounded to zero. Since the earlier publication covered the BER parameter simulations, therefore this was skipped in this article [62].

In Figure 9, which compares measured EVM values, it is noticeable that starting from approximately 30–34 dB, the adaptive algorithms positively influenced the whole simulation. In the worst-case scenario, the difference between the equalized signal and the signal without the equalizer was approximately 14% for 4-QAM or 12% for 8-QAM. The difference between individual equalization techniques increased with the higher order of QAM modulation (4-QAM =1.4%, 16-QAM =3%). The settings of the equalizer parameters can be seen in Table 1. These parameters were chosen based on extensive simulations similar to the experiments presented by Martinek et al. in 2017 and exhibited the best results across the board [63].

## 6. Measurements

**Figure 10:**
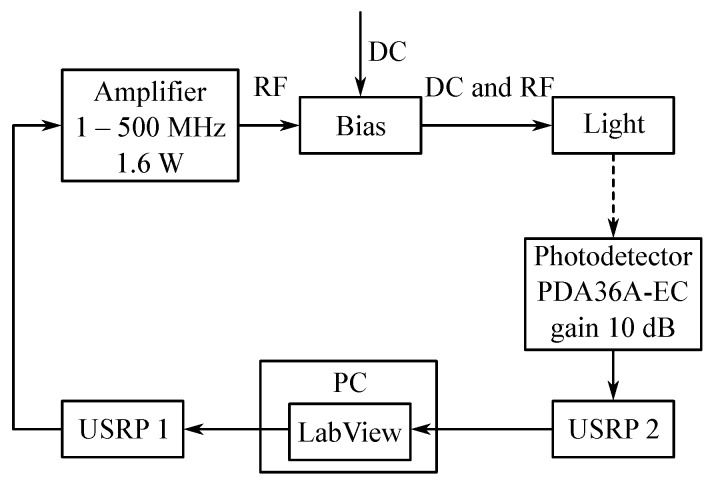
Block diagram of the tested platform.

Measurements were carried out on a custom platform developed around software defined radios (National Instruments USRPs) and virtual instrumentation (LabVIEW 2018). The Philips Fortimo LED 3000 44W/840 was used as a transmitting element. Three previously mentioned adaptive algorithms—LMS, NLMS, and QR-RLS—were used during the measurements. The monitored parameters were E${}_{b}$/N${}_{0}$, MER, EVM, and BER. The block diagram of the whole platform can be seen in Figure 10.

### 6.1. Software

As mentioned before, the software side was implemented in LabVIEW 2018. This platform offered a high level of modularity and adaptability, as the software side, which was the backbone of the whole prototype, communicated directly with the software defined radios. Therefore, any modifications of the software side could be instantaneously tested during the real measurements.

The front panel of the prototype consisted of four parts. The first part was used to set the proper signal parameters (carrier, bandwidth, USRP gain, sample width), modulation parameters (mainly QAM order), IP addresses of USRPs, and the path of the output file with the measured parameters. The second part focused on equalizations, offering variable configurable parameters, like the regularization factor, leakage, or forgetting factor. The third part or block was mainly used for measurement, offering various readable parameters (BER, MER, EVM, etc.). Blocks 4 and 5 showed the waveform graphs on the transmitting and receiving side.

### 6.2. USRP

Two NI USRP-2921 were used for the experiments (Figure 10). These older USRPs use standard Ethernet as an interface between the device itself and the controlling computer. Other than that, USB 2.0 can be also used for communication, although it offers lower bit rates. Two SMA connectors for the transmitting or receiving side were also present. Synchronization could be either software driven, driven by an external clock generator, or via bus by connecting a proprietary PCI-E like cable. It originally offered daughterboards operating at 2.4 to 2.5 GHz and 4.9 to 5.9 GHz, which were not suitable for VLC experiments. Therefore, two boards—Ettus LFTX for the transmitter and LFRX for the receiver—which operated at 0–30 MHz, were used during the experimental measurements.

### 6.3. Amplifier and Bias Tee

The amplifier constructed by Chinese engineers was used during the measurements. It operated at 1 to 500 MHz and amplified the signal by 1.6 W.

The Mini-Circuits ZX85-12G-S+ bias tee was used to modulate Philips light source. It offered a low insertion loss of 0.6 dB, wideband operation (0.2 to 12,000 MHz), a high current capability of 400 mA, and a rugged unibody construction. The bias tee itself was a diplexer, which used the low-frequency port to set the bias, and the high-frequency port passed the RF signal, but blocked the biasing. The combined port used both bias and RF to modulate the light source itself.

### 6.4. Photodetector

Thorlabs PDA36A-EC was used as a receiving element. It was a Si PIN photodetector with switchable gain operating at 350 to 1100 nm. It offered a bandwidth of up to 10 MHz and a peak sensitivity at 970 nm. Various lens tubes could be mounted onto the thread coupler to direct light at the receiving chip itself. However, during the measurements, the lenses were not used, as it was necessary to simulate the receiving elements (such as mobile phones).

### 6.5. Configuration, Scenario, and Results

The following configuration was used during the measurements: a carrier frequency of 3 MHz with a variable bandwidth from 1 MHz to 4 MHz. However, since NI USRPs had some channel widths fixed, it used a slightly wider channels (for example 3.0303 MHz instead of 3 MHz). The number of transmitted symbols was 10,000.

The measurement itself was carried out in the laboratory of the Technical University of Ostrava. The Philips Fortimo ceiling light was mounted on a moveable holder, which could be freely adjusted on a custom ceiling rail system. This type of light was chosen based on the statistics from Czech public institutions. It is often used in the construction of new buildings or during renovations of older ones. Since it was also used in the university corridors, the whole system could be beta tested directly during full operation. The selected parameters of this light source can be seen in Table 2.

**Figure 11:**
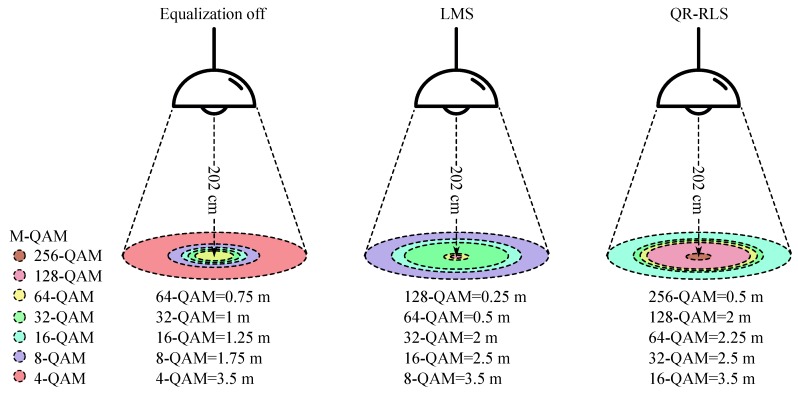
Visualization of reached M-QAM modulation formats based on measured parameters—3 MHz channel width and carrier frequency.

The receiving photodetector was mounted on a movable laboratory cart. Therefore, it was possible to change the position of the detector, but always keeping the same height. This scenario can be seen in Figure 11. The Philips light source illuminated conical area with diameter of 700 cm.

The measurement itself was carried out only in the X axis up to a maximum distance of 350 cm, which corresponded to the radius of the illuminated area (Figure 11). As the illuminated area was a symmetrical cone, the measurement was carried out only in one direction: from the center to the “right” side. Therefore, the measured values for one direction corresponded to the measurements in every other direction (this statement was tested and verified by random short measurements).

**Figure 12:**
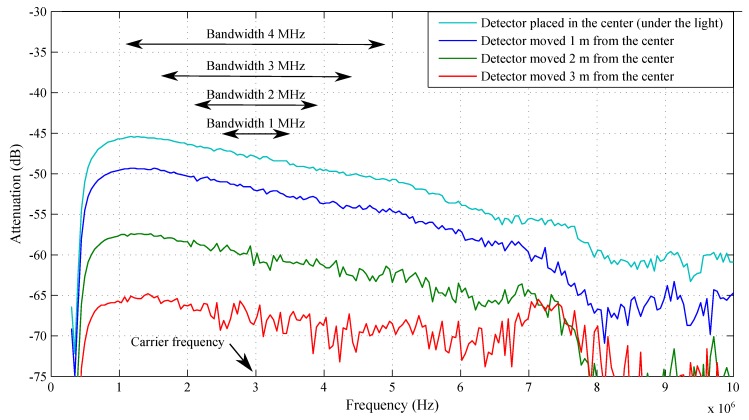
Attenuation frequency response of the entire communication chain using a Philips Fortimo LED 3000 44W/840 lamp at a distance of 202 cm between the source and the photodetector.

The Rohde & Schwarz ZVB4 network analyzer was used to measure the attenuation characteristics of the entire communication chain. This attenuation frequency response was measured against the reference that was set when the analyzer input and output were connected directly. After measuring this characteristic, it was decided that the measurement would be carried out at a carrier frequency of 3 MHz, mainly to use as much bandwidth as possible. The attenuation frequency characteristics measured at various distances (1 m, 2 m, and 3 m) from the center of the illuminated area are visible in Figure 12 as well. It is noticeable that the attenuation rapidly increased with the increasing distance from the center.

**Figure 13:**
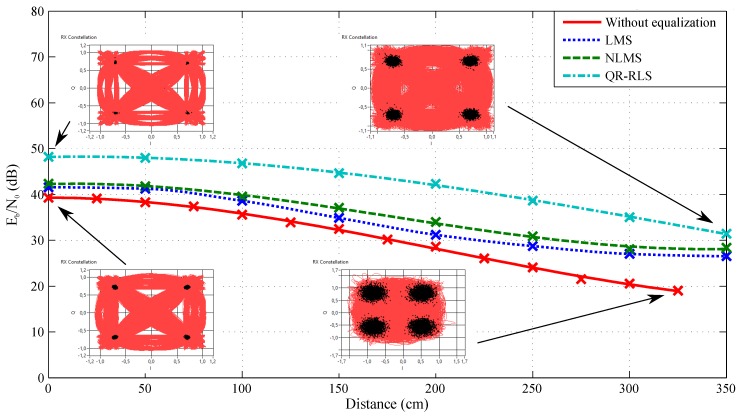
Dependence of E${}_{b}$/N${}_{0}$ on the distance and adaptive algorithm, bandwidth 1 MHz, 4-QAM.

**Figure 14:**
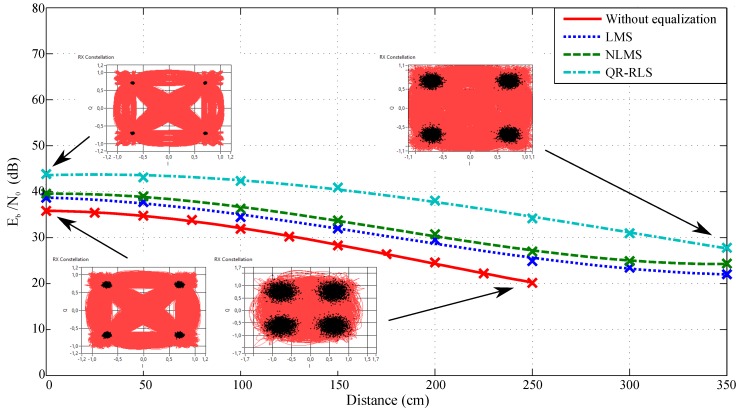
Dependence of E${}_{b}$/N${}_{0}$ on the distance and adaptive algorithm used, bandwidth 4 MHz, 4-QAM.

The dependence of E${}_{b}$/N${}_{0}$ on the measured distance and configured adaptive algorithm can be seen in Figure 13 and Figure 14. These figures correspond to measurements with 4-QAM modulation and the 1 MHz or 4 MHz bandwidth. It is noticeable that the QR-RLS adaptive algorithm provided the best results of E${}_{b}$/N${}_{0}$ across the board (in comparison to the non-equalized data). The parameter was improved by 9 dB at the center of the illuminated area and by 11 dB at a threshold value of almost 300 cm. The other algorithms improved the measured signal as well, but the difference was lower. LMS managed to improve the E${}_{b}$/N${}_{0}$ by 2 dB at the center and 7 dB at the threshold. NLMS improved the values by 3 dB at the center and 8 dB at the threshold. As is visible, each algorithm noticeably improved the received signal.

These figures also include two sets of constellation diagrams, one for the scenario without equalization and one with the QR-RLS adaptive algorithm. Each set had two constellation diagrams corresponding to the values in the center of the illuminated area and at the measurable threshold (350 cm for QR-RLS and 325 cm for the scenario without equalization). By comparing both figures, it can be seen that wider bandwidths had a significant impact on E${}_{b}$/N${}_{0}$. Figure 13 shows the better results of E${}_{b}$/N${}_{0}$ by approximately 4 dB in comparison to Figure 14. However, wider channels also offered higher transmit speeds.

**Figure 15:**
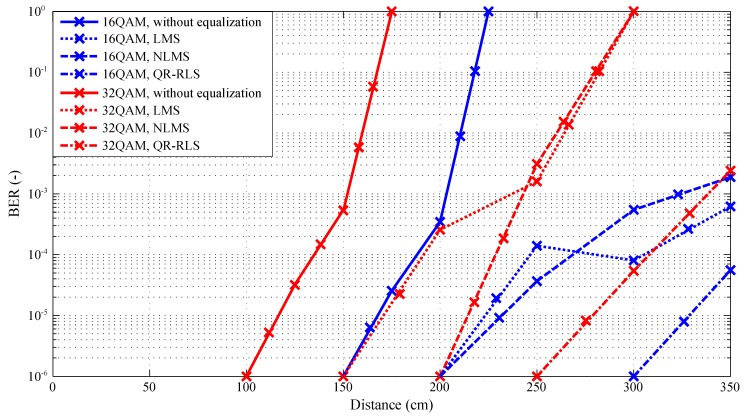
Dependence of BER on the distance and used adaptive algorithm, bandwidth 1 MHz, 4–32-QAM.

**Figure 16:**
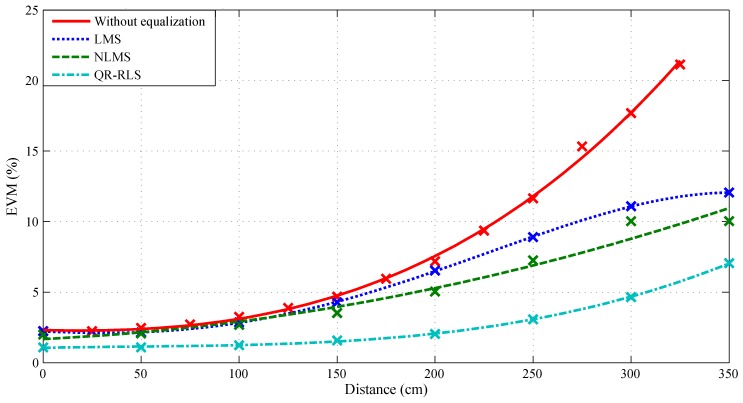
Dependence of EVM on the distance and used adaptive algorithm, bandwidth 1 MHz, 4-QAM.

Figure 15 and Figure 16 show the comparison of the BER and EVM parameters. Both figures displayed a bit error rate for the 1 MHz bandwidth and 4–32 QAM modulation formats. Some waveforms are not present in the figures, as they tended to copy the horizontal axis (their BER was out of the platform measuring range). This fact mainly affected the 4-QAM modulation scheme in combination with various adaptive algorithms and was caused by the limited number of transmitted symbols, effectively influencing the range of the measurable bit error rate. Modulated signals without equalization had significantly higher error rates. The 32-QAM modulation scheme at 300 cm never exceeded the order of ${}^{-5}$, when the QR-RLS algorithm was used. In comparison, the whole platform easily exceeded the order of ${}^{-4}$ at 150 cm without any equalization algorithms. The 4-QAM modulation would need to be used to reach a distance of 300 cm without equalization algorithms; therefore, the effective transmit speed would be lowered significantly. The system with this configuration might be able to transmit even on 325 cm, but the BER decreased rapidly (${}^{-4}$ at 325 cm).

The EVM comparison in Figure 13 shows that the QR-RLS exhibited the best results. QR-RLS managed to improve EVM by 1.4% at the center of the illuminated area and by up to 10% at 300 cm. LMS and NLMS had very similar results: LMS improved EVM by 0.02% at the center and 5% at 300 cm, while NLMS improved EVM by 0.25% at the center and 7% at 300 cm.

All the above figures show that QR-RLS appeared to be the best adaptive algorithm, improving the measured parameters significantly. It was followed by NLMS and LMS, which had very similar results. Part of the results can be seen in Table 3 and Table 4.

## 7. Discussion

**Figure 17:**
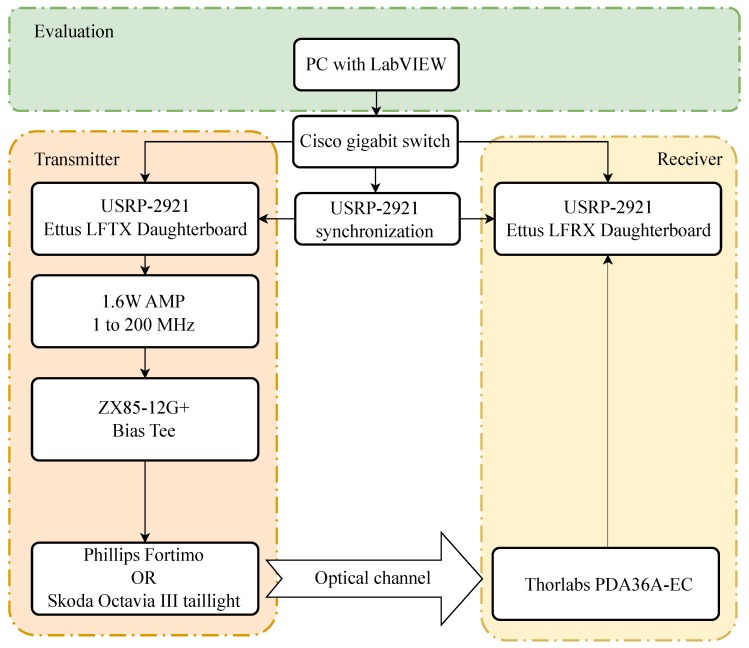
Schematics of the developed beta OFDM system.

While the presented equalization algorithms definitely improved the measured parameters, there was still room for improvement. The system vastly improved the first generation of our VLC system, mentioned previously. However, QAM in this field is slowly pushed back in favor of more modern OFDM variants. Therefore, during the testing, the development of the beta OFDM system, already began (Figure 17). It was based on modified hardware components from the presented QAM system. During the development, a third USRP had to be used, since the OFDM was highly sensitive to precise synchronization. However, a limitation of gigabit Ethernet was reached as well; therefore, the whole platform would be modernized and moved to newer SDRs with the PCI-Express interface.

Based on the measurements, the used amplifier suffered from significant non-linearity. Alternative models are currently being explored, and LZY-22+ from Mini Circuits is the most likely candidate for future testing. Non-linearity significantly influenced the results, and the values might be even improved by at least 30%.

A number of filters could be also tested in future works, as indicated by Tokgoz et al. [64], who tested blue and flattening filters. Their system managed to improve BER values by 15–40%. According to them, blue filters omitted about 60% of the LED transmit power, while the response-flattening filter did not influence the LED power capabilities. To test further revisions of the presented platform, a set of blue light filters from Thorlabs was acquired. Further tests are scheduled for newly developed OFDM systems.

The receiver was another limiting factor of the whole platform. The commercially available Thorlabs solutions suffer from a lower quality of in-built amplifier. To overcome this issue, a set of PIN photodiodes from Hamamatsu photonics was acquired, and a new, custom PIN photodetector is planned. A test APD based photodetector is also planned. The solution from Hamamatsu seems to be the most suitable candidate, and its acquisition is a topic of future cooperation.

A new set of adjustable lenses was also acquired from Thorlabs. The previously used lens was scratched upon the final set of measurements, so the results were scrapped. However, replacements parts are already scheduled to arrive soon.

The final step will be an implementation of an FPGA based version. The platform at it stands is highly dependent on the performance of the computer, which is used for LabVIEW code execution. It is basically a tradeoff between modularity and development speed vs. platform speed. The current implementation is significantly slower than the FPGA version, but any adjustments can be quickly tested and pulled back if needed.

**Figure 18:**
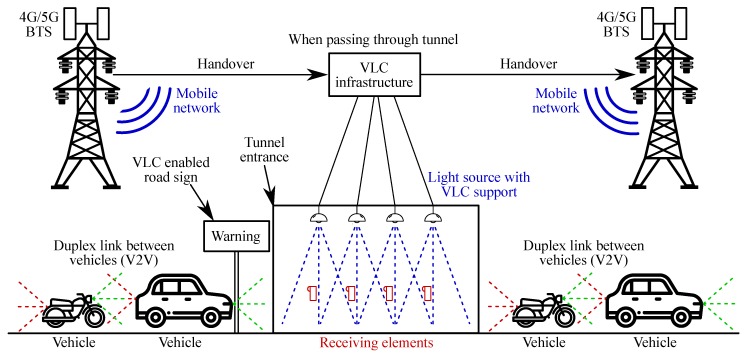
Proposed concept of the hybrid VLC-4G/5G network for vehicular data transmissions.

The presented results can be transferred to car light sources, as the presented platform is very modular and offers a certain degree of portability. Further tests of modulation schemes in outdoor environments are planned, as well as compatibility with modern mobile networks. Figure 18 shows an early concept of a hybrid 4G/5G/VLC network for communication in congested environments. Mobile network base stations would handover connections to the VLC infrastructure in certain advantageous scenarios (such as road tunnels). After the vehicle passes through the congested environment, the VLC infrastructure passes the user equipment back to the mobile network. VLC technology can be also used for tarification based on the VIN of individual vehicles.

Avatamanitei et al. presented their own platform for vehicular VLC. Their system was built around traffic lights and provided a noise resilient solution. It was capable of communication up to 50m, which was similar to the preliminary results of our OFDM based platform. OFDM/QAM definitely offered higher transmit speeds in comparison to OOK; however, this experiment mainly tested the influence of direct sunlight. The presented method is very interesting and will definitely be a topic for future research [65].

Further experiments will be also carried out in the BROADBAND${}^{\mathrm{LIGHT}}$ testing polygon situated next to the Faculty of Electrical Engineering and Computer Science at the Technical University of Ostrava. It consists of a full-mesh network solution, covering 10 lamp posts, which altogether carry 18 LED public lighting sources from various manufacturers. Each lamp can be individually and remotely controlled from the lab. The setup is prepared for native deployment of VLC and serves as a real-life showroom of smart technologies.

Another set of similar tests will be carried out on the Octavia III taillight. For this different light source, an approach previously used in an earlier article will be used. A special box used for the simulation of various natural phenomena will be used to simulate the ever-changing conditions of weather throughout the year.

The development of new custom parts is currently ongoing. Apart from the photodetector mentioned earlier, a new and less expensive bias tee was constructed. This new design should push manufacturing costs lower, while maintaining a certain level of quality. Moreover, it is much easier to replace damaged parts on site, as it is possible to avoid external manufacturing. A new custom light source with interchangeable LED matrices is also under development to test the various influences of LED quality on VLC [66].

## 8. Conclusions

This work dealt with channel feed-forward software defined equalization in a QAM VLC system. The whole platform based on LabVIEW and National Instruments hardware was introduced. The first part presented the results from simulation software, which roughly estimated the expected results. QR-RLS was preliminarily identified as the best algorithm for deployment; however, this argument had to be backed by certain data.

The second part described the whole measuring phase, which backed up the earlier statement. QR-RLS managed to improve BER values by up to one whole order. It also improved EVM up to 10%. LMS and NLMS improved the measured values as well, but the impact was lower. NLMS managed to improve EVM up to 7% in the best-case scenario, whereas LMS reached only up to 5%.

Certain weak spots were identified and discussed. Further research was outlined with the focus on new parts, which will significantly influence the performance of future platforms. 

## Figures and Tables

**Table 1:** Equalizer settings.

Adaptive Algorithm	QAM	Filter Length	Regularization Factor	Forgetting Factor
LMS	4 to 64	33	0.009	–
NLMS	4 to 16	33	0.09	–
NLMS	32 to 64	33	0.3	–
QR-RLS	4 to 64	34	-	0.999

**Table 2:** Parameters of the transmitting LED light source.

Philips Fortimo LED 44W/840	
Luminous power	3000 lm
Active power	46 W
Voltage	220–240 V
Effectivity	68 lm/W

**Table 3:** Table of the measured parameters of the ceiling light, 4-QAM modulation format, bandwidth 1 MHz.

**4-QAM**									
**Without EQ**					**LMS**				
**Distance**	**E${}_{b}$/N${}_{0}$**	**EVM**	**BER**	**MER**	**Distance**	**E${}_{b}$/N${}_{0}$**	**EVM**	**BER**	**MER**
**(cm)**	**(dB)**	**(%)**	**(-)**	**(dB)**	**(cm)**	**(dB)**	**(%)**	**(-)**	**(dB)**
0	39.29	2.25	NaN	32.97	0	41.56	2.23	NaN	33.35
50	38.27	2.47	NaN	32.13	50	41.25	2.15	NaN	33.05
100	35.58	3.25	NaN	29.75	100	38.61	2.86	NaN	30.89
150	32.46	4.68	NaN	26.59	150	34.88	4.28	NaN	27.37
200	28.61	7.19	NaN	22.86	200	31.20	6.53	NaN	23.70
250	24.08	11.65	NaN	18.68	250	28.73	8.90	NaN	21.01
300	20.56	17.69	NaN	15.05	300	27.03	11.09	NaN	19.10
350	19.02	21.14	4.26${}^{-4}$	13.50	350	26.54	12.06	NaN	18.37
**4-QAM**									
**NLMS**					**QR-RLS**				
**Distance**	**E${}_{b}$/N${}_{0}$**	**EVM**	**BER**	**MER**	**Distance**	**E${}_{b}$/N${}_{0}$**	**EVM**	**BER**	**MER**
**(cm)**	**(dB)**	**(%)**	**(-)**	**(dB)**	**(cm)**	**(dB)**	**(%)**	**(-)**	**(dB)**
0	42.35	2.00	NaN	33.98	0	48.21	1.09	NaN	39.46
50	41.79	2.07	NaN	33.68	50	47.96	1.09	NaN	39.25
100	39.54	2.68	NaN	31.45	100	46.78	1.24	NaN	38.13
150	37.10	3.52	NaN	29.08	150	44.65	1.58	NaN	36.03
200	33.99	5.05	NaN	25.93	200	42.32	2.04	NaN	33.80
250	30.87	7.25	NaN	22.79	250	38.65	3.08	NaN	30.23
300	28.07	10.03	NaN	19.98	300	35.03	4.66	NaN	26.64
350	28.37	10.04	NaN	19.97	350	31.42	7.05	NaN	23.03

NaN (not a number)represents immeasurable BER (values below 10${}^{-5}$ threshold).

**Table 4:** Table of measured parameters of the ceiling light, 256-QAM modulation format, bandwidth 1 MHz.

**256-QAM**									
**Without EQ**					**LMS**				
**Distance**	**E${}_{b}$/N${}_{0}$**	**EVM**	**BER**	**MER**	**Distance**	**E${}_{b}$/N${}_{0}$**	**EVM**	**BER**	**MER**
**(cm)**	**(dB)**	**(%)**	**(-)**	**(dB)**	**(cm)**	**(dB)**	**(%)**	**(-)**	**(dB)**
0	23.98	5.32	3.87${}^{-2}$	21.21	0	32.69	1.90	1.00${}^{-3}$	30.18
50	—	—	—	—	50	30.09	3.00	3.03${}^{-2}$	27.50
100	—	—	—	—	100	29.49	2.78	6.38${}^{-3}$	26.90
150	—	—	—	—	150	23.25	6.16	9.16${}^{-2}$	20.39
200	—	—	—	—	200	—	—	—	—
250	—	—	—	—	250	—	—	—	—
300	—	—	—	—	300	—	—	—	—
350	—	—	—	—	350	—	—	—	—
**256-QAM**									
**NLMS**					**QR-RLS**				
**Distance**	**E${}_{b}$/N${}_{0}$**	**EVM**	**BER**	**MER**	**Distance**	**E${}_{b}$/N${}_{0}$**	**EVM**	**BER**	**MER**
**(cm)**	**(dB)**	**(%)**	**(-)**	**(dB)**	**(cm)**	**(dB)**	**(%)**	**(-)**	**(dB)**
0	32.10	1.99	1.48${}^{-3}$	29.78	0	40.62	0.79	0	37.81
50	30.60	2.42	3.74${}^{-3}$	28.12	50	40.96	0.75	0	38.28
100	27.76	3.42	1.15${}^{-2}$	25.15	100	38.91	0.94	1.70${}^{-5}$	36.33
150	22.86	6.39	6.71${}^{-2}$	20.03	150	35.55	1.40	1.15${}^{-4}$	32.88
200	—	—	—	—	200	28.00	3.66	5.70${}^{-2}$	25.18
250	—	—	—	—	250	20.70	8.53	1.57${}^{-1}$	17.83
300	—	—	—	—	300	—	—	—	—
350	—	—	—	—	350	—	—	—	—

— Values could not be measured due to signal quality.
